# Oxidation leaching of chromium from electroplating sludge: Ultrasonic enhancement and its mechanism

**DOI:** 10.3389/fchem.2022.958773

**Published:** 2022-08-25

**Authors:** Kaihua Huang, Lizhangzheng Wang, Yong Wen, Kuang He, Mingyang Zhang, Jianwei Du, Xiaoying Hu

**Affiliations:** ^1^ South China Institute of Environmental Sciences, Ministry of Ecology and Environment, Guangzhou, China; ^2^ School of Resources and Environmental Engineering, Wuhan University of Technology, Wuhan, China

**Keywords:** chromium, oxidation leaching, ultrasonic enhancement, electroplating sludge (EPS), mechanism

## Abstract

The oxidation leaching of chromium from electroplating sludge was investigated, and ultrasonication was introduced for the enhancement of the leaching process. Two different types of Cr-bearing electroplating sludge were selected for the study, and the effects of the reagent dosage, temperature, and ultrasonic pulse ratio on the leaching efficiency were tested through oxidation leaching experiments. The experimental results show that hydrogen peroxide and sodium hypochlorite exhibit different leaching effects on different types of electroplating sludge. The control of reagent dosage is crucial for the oxidation leaching of Cr, while the effect of temperature turns out to be small. Hydrogen peroxide turns out to be a more effective oxidizer for chromium sludge, and the leaching efficiency of Cr could be promoted from 77.52% to 87.08% using ultrasonic enhancement under optimum conditions. Interestingly, sodium hypochlorite exhibited better leaching efficiency than hydrogen peroxide for the mixed sludge since the organic matter in the mixed sludge will lead to the rapid decomposition and consumption of hydrogen peroxide. The leaching efficiency of Cr from the mixed sludge could also be promoted from 56.82% to 67.10% using ultrasonic enhancement under optimum conditions. According to the scanning electron microscope imaging, ultrasonic enhancement can create voids and cracks on the surface of the sludge particles, hence promoting the contact between electroplating sludge and leaching agents, and promoting the oxidation leaching efficiency. In addition, ultrasound seems to be able to remove the coverings on the surface of the mixed sludge particles, which may facilitate the oxidation reaction.

## 1 Introduction

Electroplating sludge (EPS) is the heavy metal-containing sludge produced after the treatment of wastewater in the operation of electroplating industry. Generally, electroplating sludge can be divided into two categories, including separated sludge and mixed sludge. Separated sludge contains single heavy metal element, such as copper sludge, nickel sludge, and chromium sludge. Mixed sludge contains two or more heavy metal elements and is usually mixed with organic sludge such as oily sludge and biochemical sludge. Since the heavy metals in EPS pose great risks to environment and human health, EPS has been listed as hazardous waste in many countries and regions, including China and the European Union (Magalhaes et al., 2005). What’s more, with the development of machinery manufacturing industry and advances in technology like composite coating (Mahmoudi et al., 2021; Shakiba et al., 2022), the amount of chromium coating and demand for chromium resources are increasing, which makes the resource utilization of chromium-bearing wastes become even more important. Thus, how to deal with electroplating sludge reasonably and effectively has attracted the attention of many research workers.

At present, the main treatment methods for EPS include harmless treatment and resource utilization. Harmless treatment methods mainly include stabilization (Chen et al., 2020) and incineration (Zhou et al., 2018). However, with the increasing requirements for environmental protection and carbon emissions, resource utilization has become a more preferred method than harmless treatment, especially for the treatment of wastes with a high utilization value such as EPS. The resource utilization methods for electroplating sludge mainly include pyrometallurgy (Huang et al., 2013), hydrometallurgy (Wu et al., 2020), and preparation of functional materials like construction materials, catalysts, and pigments (Carneiro et al., 2018; Zhang et al., 2018; Dai et al., 2019). Although the preparation of functional materials from EPS is more value-added, the application of the product is usually restricted, which limits the applicability of the technology. Therefore, pyrometallurgy and hydrometallurgy methods are now more widely used in the industry.

The pyrometallurgy method is more suitable for the treatment of separated sludge with high content of Cu or Ni, while Cr cannot be effectively recovered since the melting point of Cr is much higher. Commonly, acid leaching is employed for extracting heavy metals from mixed EPS due to its simplicity and convenience (Yan et al., 2019). Nevertheless, the acid leaching method is nonselective for mixed heavy metal sludge, resulting in multi-metal solutions, which require further separation operations (Li et al., 2010). Solvent extraction is usually applied for the separation of mixed heavy metals from the acid leachate, but its procedure is relatively complicated (Kul and Oskay, 2015). Currently, the electrowinning method has been developed to selectively recover high-purity Cu from the acid leaching solution of electroplating sludge containing Cu and Ni (Veglio et al., 2003; Wang et al., 2018), while Cr plays a negative role in the process which will hinder the utilization of the multi-heavy metal solutions. Therefore, the selective recovery of Cr from electroplating sludge is necessary, but research in this area is scarce (Yue et al., 2019; Zheng et al., 2020). Existing research shows that Cr can be selectively recovered by Cr (III) oxidation under high temperature roasting and the subsequent Cr (VI) leaching; the leaching rate of Cr (IV) can reach higher than 90% (Ding et al., 2008; Verbinnen et al., 2013). However, the high temperature roasting process is accompanied by high energy consumption and smoke pollution. Thus, the oxidation of Cr (III) under low temperature is a promising idea for the selective recovery of Cr from EPS, which could avoid high energy consumption and smoke pollution. Research workers have studied the oxidation leaching of molybdenum from molybdenite concentration with different oxidizing agents under lower temperature, and 89.3% of the Mo-leaching rate was achieved under optimum conditions, which provides a good guide for our research (Arbat et al., 2020).

In this study, the oxidation leaching of chromium from chromium sludge and mixed sludge was investigated and compared, which would provide a more comprehensive perspective for the resource utilization of electroplating sludge. The difference between the two kinds of sludge was analyzed, including physical, chemical, and reaction properties. The effects of different reaction conditions on the leaching results were investigated and ultrasonication was introduced to enhance the oxidation leaching process. Finally, the surface morphology change of EPS was investigated by a scanning electron microscope to explain the influence mechanism of the leaching process. This study provides a novel thought for the selective extraction of chromium from Cr-bearing EPS; the findings of this study may give significant guidance for the resource utilization of Cr-bearing EPS.

## 2 Materials and methods

### 2.1 Material

Two different types of Cr-bearing electroplating sludge were collected for the study, including the chromium sludge and the mixed sludge.

The chromium sludge (EPS1) used in this study was collected from a chromium plating company in Guangzhou city (China), where chromium-containing wastewater is precipitated by sodium hydroxide after reduction with a reducing agent and then filtered by a pressure filter separately. Thus, EPS1 mainly consists of Cr(OH)_3_ and inorganic matters.

The mixed sludge (EPS2) was collected from the wastewater treatment plant of an electroplating park in Jiangmen city (China), where different types of sludges were mixed and filtered, including chromium sludge, copper sludge, nickel sludge, and oily sludge. Thus, EPS2 mainly consists of different types of heavy metal hydroxides and organic matters.

Prior to use, the sludge was dried at 105°C to constant weight. The sample was all ground by milling and sieved with a particle size of ≤150 μm to get the prepared sludge power. The prepared sludge powder was then used in the following oxidation leaching experiments.

Other chemicals used in this study were purchased from commercial suppliers with analytical-grade purity. All experiments were carried out with distilled water.

### 2.2 Oxidation leaching

The oxidation leaching experiments were carried out in water bath under stirred condition. One gram of the prepared EPS sample was weighed and added into a 50-ml beaker. A certain volume of 2 mol/L sodium hydroxide solution was then added, followed by a certain amount of oxidizer, and finally, an appropriate amount of pure water was added to control the liquid–solid ratio of about 20:1 for the reaction. The water bath temperature was set to a desired value, and the reaction lasted for 2 h. After the reaction, the leachate was filtered and separated; the metal content in the leachate was determined by an inductively coupled plasma mass spectrometer (ICP-MS, 7800 ICP-MS system, Agilent Technologies Co., Ltd., United States) or an atomic absorption spectrometer (AAS, PinAAcle 900T, PerkinElmer, United States), and the residue was weighed after drying.

The ultrasonic-enhanced leaching experiment was carried out in the ultrasonic cell crusher (SM-900A, Shunmatech, Nanjing). The reactants were mixed in the beaker as described earlier, then the ultrasonic horn was placed in the beaker below the liquid level, and the ultrasonic action time, frequency, power, and pulse ratio were adjusted to the desired value before the reaction began. After the reaction, the metal content in the leachate was determined by the same method.

### 2.3 Characterization

The organic content of the EPS was tested by the gravimetric method as described in the relevant industry standard (CJ/T 221-2005). The crystal mineralogical composition of the prepared sludge powder was analyzed by X-ray diffraction (XRD, X’pert Pro MRD, PANalytical BV, the Netherlands). The chemical composition of the sludge powder was determined using ICP-MS after digestion using an HF-HNO_3_-HCl mixed solution. The surface micro-topography of the sludge powders before and after oxidation leaching were characterized by a field emission scanning electron microscope (SEM, GeminiSEM300, ZEISS, Germany) and an electron probe micro-analysis (JXA-8100, JEOL, Japan).

Fraction analysis of heavy metals present in the sludge was carried out by a modified Community Bureau of Reference (BCR) three-stage sequential extraction procedure; the details of the extraction steps are as described by Rauret et al. (1999). According to the method, metal forms in the sludge were categorized into four fractions: acid soluble fraction, reducible fraction, oxidizable fraction, and non-mobile residual fraction.

## 3 Results and discussion

### 3.1 Characterization of electroplating sludge

The content of organic matter and main metal elements in chromium sludge (EPS1) and mixed sludge (EPS2) is shown in Table 1. The results show that the composition of EPS1 is relatively simpler than that of EPS2. EPS1 has very high content of Cr of 35.96% and relatively low content of organic matter and other heavy metals. In comparison, the organic content of EPS2 is much higher than that of EPS1, and EPS2 contains multiple heavy metal elements of contents ranging from 1% to 6%.

**TABLE 1:** Main composition of the electroplating sludge.

Sample	Organic content (%)	Ca (mg/kg)	Cr (mg/kg)	Fe (mg/kg)	Ni (mg/kg)	Cu (mg/kg)	Zn (mg/kg)
EPS1	4.19	3,313.4	359,621.3	846.2	9,298.5	5,575.9	28.2
EPS2	16.12	90,320.4	21,589.9	27,725.6	35,224.3	56,928.9	16,218.6

**FIGURE 1:**
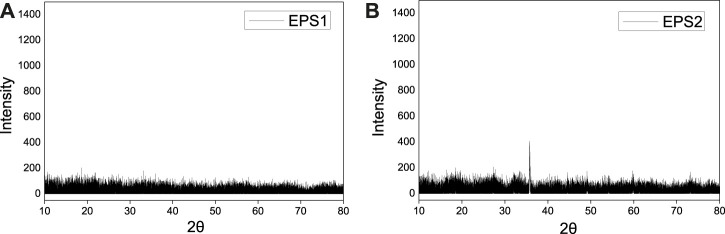
XRD pattern of **(A)** EPS1 and **(B)** EPS2.

The XRD patterns of the two sludge powders are shown in Figure 1, which indicated that the heavy metal compounds in the sludge did not form crystalline matters for both samples. The amorphous state of the EPS samples may result from the formation conditions of electroplating sludge by rapid neutralization or precipitation under room temperature and atmospheric pressure (Yang et al., 2015).

**FIGURE 2:**
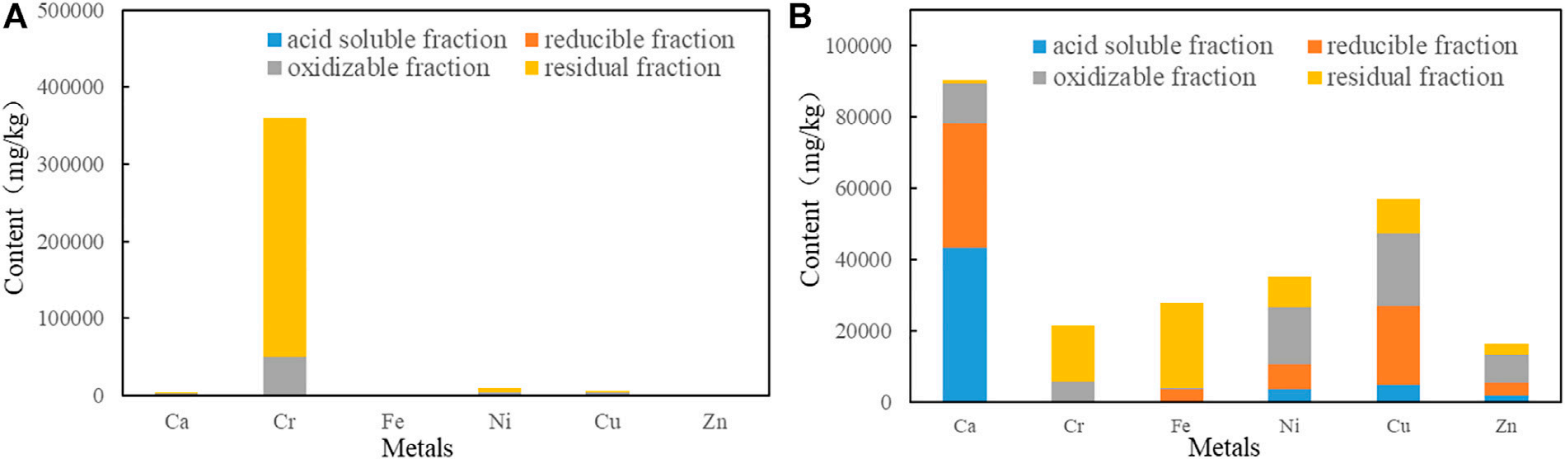
Fractions of Ca, Fe, Cu, Ni, Zn, and Cr by BCR analysis in **(A)** EPS1 and **(B)** EPS2.

Data obtained by BCR analysis (Figure 2) revealed that the dominant existence form of Cr were the oxidizable and residual fractions for EPS1 and EPS2, accounting for >99.0%. For mixed sludge (EPS2), Cu, Ni, and Zn were distributed in all four fractions. Typically, metals in the acid soluble fraction (consisting of adsorbed ions on ion-exchangeable phases) and reducible fraction (iron and manganese oxyhydroxides) are considered to be more mobile and dangerous than those in other forms, whereas metals in the oxidizable (sulfides and organic matter-bound fraction) and residual (associated with stable minerals such as silicates and crystallized oxides) fractions are considered to have lower mobility (Nguyen et al., 2015). Therefore, the high proportion of Cr in the oxidizable and residual fractions showed that Cr in sludge was more stable than Cu, Ni, and Zn (Zhang et al., 2020). The proportion of Cu, Ni, and Zn in the oxidizable fraction mainly exists in organic matter-bound form and will be released under the oxidizing condition; thus, proper alkaline condition is required for the sediment of the released ions to ensure the selective extraction of Cr.

### 3.2 Oxidation leaching of chromium sludge

Hydrogen peroxide (H_2_O_2_) and sodium hypochlorite (NaClO) are used as oxidants for the oxidation leaching of electroplating sludge under alkaline conditions. The reaction equations are shown in Eqs 1,2:

$$2\text{Cr}{\left(\text{OH}\right)}_{3}+4\text{NaOH}+3{\text{H}}_{2}{\text{O}}_{2}\overset{\;\;\;\;\;\;\;\;}{\to }2Na_{2}CrO_{4}+8{\text{H}}_{2}\text{O}$$ (1)

$$2\text{Cr}{\left(\text{OH}\right)}_{3}+4\text{NaOH}+3\text{NaClO}\overset{\;\;\;\;\;\;\;\;}{\to }2Na_{2}CrO_{4}+3\text{NaCl}+5{\text{H}}_{2}\text{O}$$ (2)

The effects of oxidizer dosage, NaOH dosage, and temperature on the leaching results were investigated by condition experiments.

**FIGURE 3:**
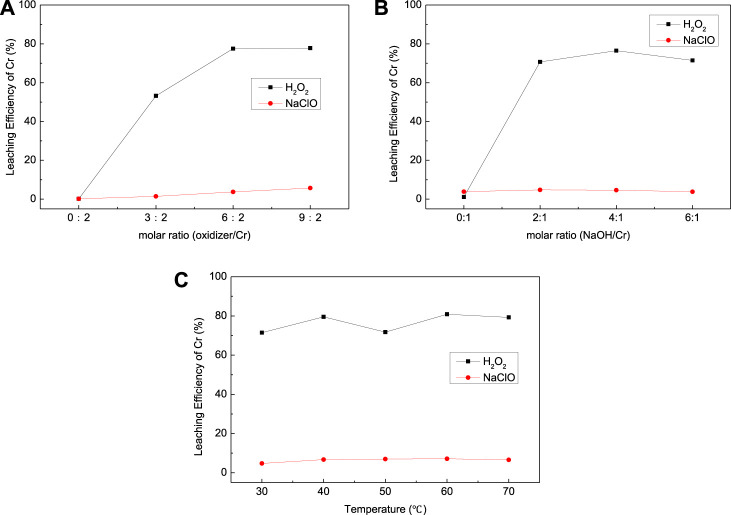
Effect of **(A)** oxidizer dosage, **(B)** NaOH dosage, and **(C)** temperature on the leaching results for chromium sludge (EPS1).

As shown in Figure 3A, the type of oxidizer has a great impact on the leaching efficiency of Cr for EPS1; H_2_O_2_ exhibited stronger leaching ability than NaClO for the oxidation leaching of Cr from EPS1. The reason is that H_2_O_2_ has higher oxidation potential than NaClO, so it possesses stronger oxidation capacity and reactivity (Behin et al., 2017). The dosage of H_2_O_2_ was about twice the reaction ratio (6:2) when the reaction reached equilibrium with Cr leaching efficiency of 77.79%; the extra consumption of H_2_O_2_ may be due to its tendency to break down during the reaction.

The effect of NaOH dosage and temperature on the leaching efficiency of Cr was also studied as shown in Figures 3B,C, respectively. As indicated by Eqs 1,2, the oxidation leaching process requires consumption of NaOH. Thus, Cr cannot be oxidized and leached without the addition of NaOH, and the increase of NaOH would increase the extraction of Cr. However, when the dosage of NaOH exceeds the molar ratio of the reaction (2:1), further increase of NaOH dosage can hardly improve the leaching efficiency. Temperature has little effect on the leaching results, which indicates that the leaching process can be operated under room temperature.

The experimental results above indicated that the oxidation leaching of Cr from EPS must be conducted under alkaline condition, whereas the BCR analysis was conducted under acidic condition. Hence, the oxidizable fraction of Cr by BCR analysis was much lower than the proportion of Cr that can be extracted by oxidation leaching. In other words, the residual fraction of Cr can also be extracted by oxidation leaching under alkaline condition.

**FIGURE 4:**
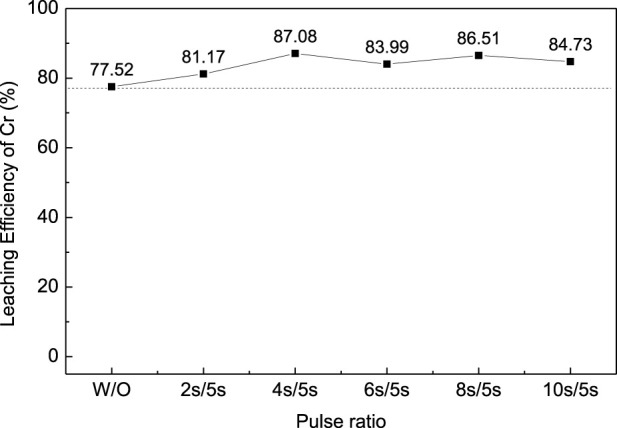
Effect of ultrasonic pulse ratio on the leaching efficiency of Cr for chromium sludge (EPS1).

In order to improve the leaching efficiency of Cr, the ultrasonic-enhanced oxidation leaching experiment was carried out under optimal conditions. The ultrasonic power was set at 450W, and the frequency was 20–25kHz; the reaction time was controled at 10 min to avoid overheating. The effect of ultrasonic pulse ratio on the leaching efficiency of Cr was studied as shown in Figure 4 (pulse ration W/O means without ultrasonic enhancement; pulse ration 2s/5s means ultrasonic enhancement intervals—on for 2s and off for 5s). The results show that the introduction of ultrasonication can improve the leaching efficiency of Cr from chromium sludge and reduce the reaction time. A pulse ratio of 4s/5s was found to be appropriate for the ultrasonication since further increase of the pulse ratio has an adverse effect on the leaching efficiency.

**FIGURE 5:**
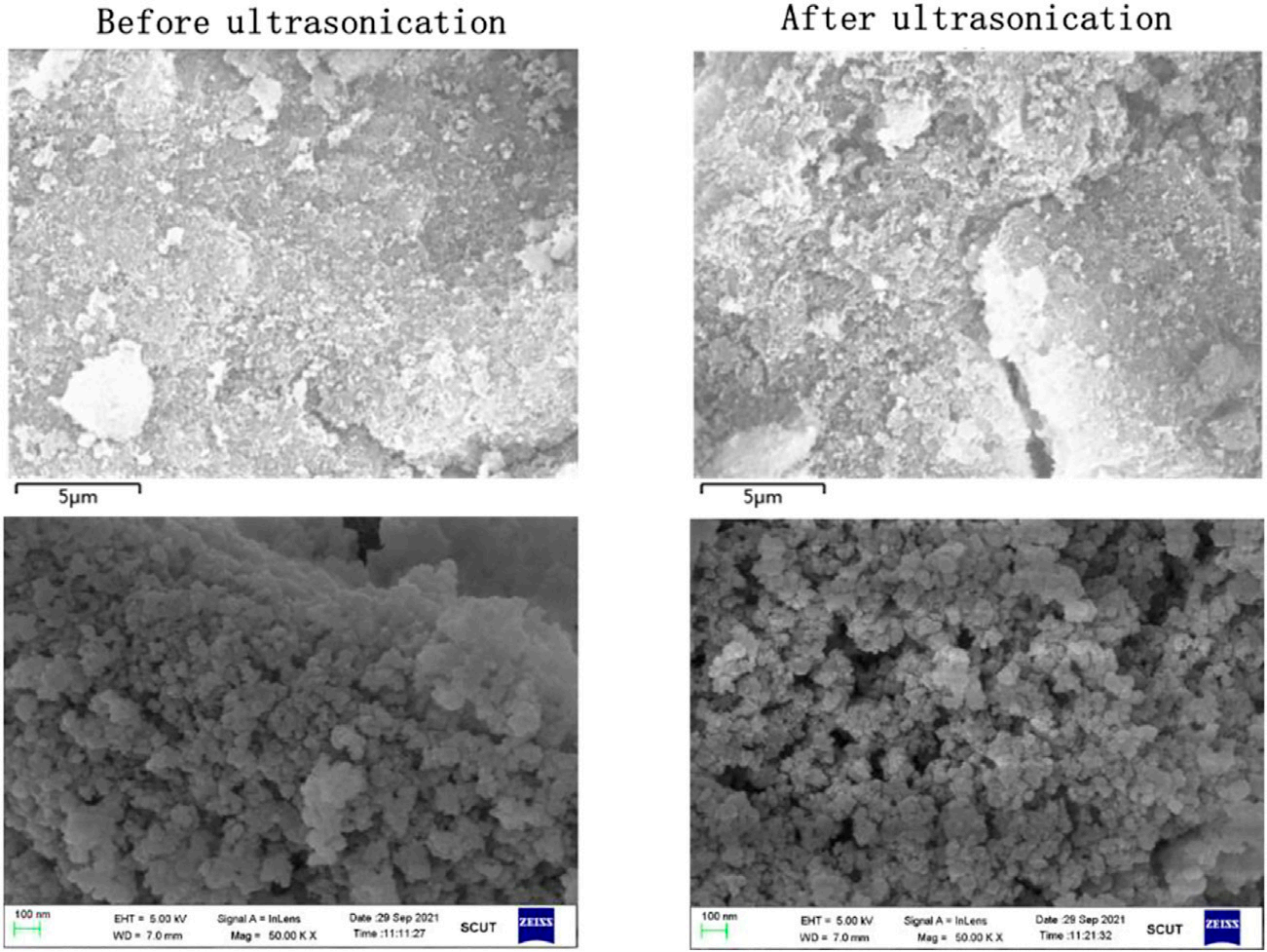
Topography change of chromium sludge (EPS1) before and after ultrasonic treatment.

**FIGURE 6:**
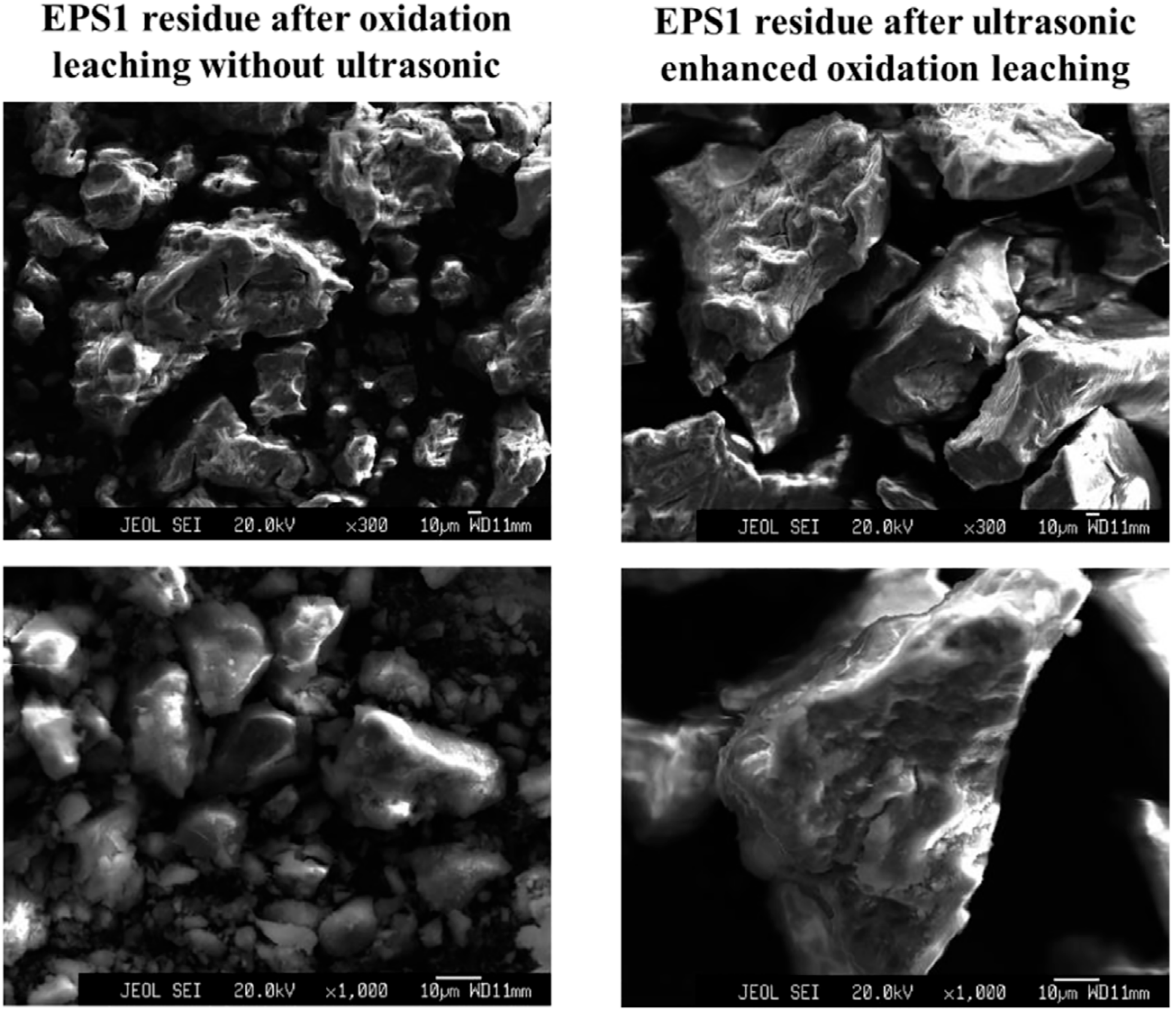
Particle morphology of chromium sludge (EPS1) after oxidation leaching with and without ultrasonic treatment.

Figure 5 shows the surface morphology changes of sludge particles before and after ultrasonic treatment at 50,000 times magnification. It can be found that there are more cracks and pores on the surface of the particles after ultrasonic treatment, thus improving the surface area of the particles. As a result, the sludge particles can react with the leaching agent more fully to improve the leaching efficiency (Zhang et al., 2017). Figure 6 shows the particle morphology of EPS1 after oxidation leaching with and without ultrasonic treatment at smaller magnification (3,000 times). The results indicated that the overall particle size of sludge particles increased after ultrasonic-enhanced leaching, which means the ultrasonication may cause particle agglomeration (Spengler and Jekel, 2000). Therefore, it is deduced that the influence of ultrasonic enhancement on the leaching process was a result of the combined effect of particle surface destruction and particle agglomeration. The extraction of Cr was substantially enhanced when ultrasonication was introduced since the effect of particle surface destruction plays a more critical role. However, when the ultrasonic pulse ratio exceeds a certain value, the effect of particle agglomeration will become stronger, leading to the decrease of leaching efficiency, which is consistent with the experimental results shown in Figure 4.

### 3.3 Oxidation leaching of complex sludge

**FIGURE 7:**
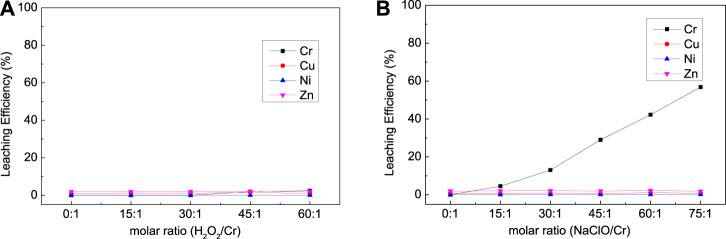
Effect of oxidizer dosage on the leaching results [**(A)** with H_2_O_2_; **(B)** with NaClO] for complex sludge (EPS2).

**FIGURE 8:**
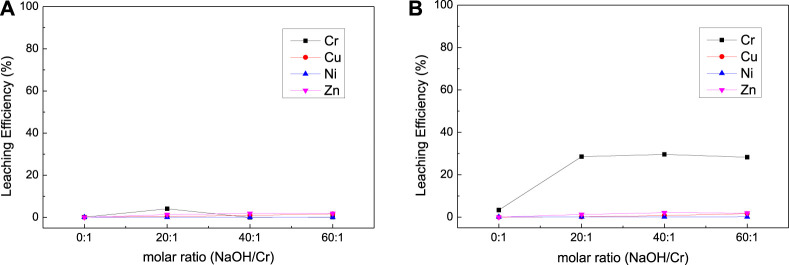
Effect of NaOH dosage on the leaching results [**(A)** with H_2_O_2_; **(B)** with NaClO] for complex sludge (EPS2).

**FIGURE 9:**
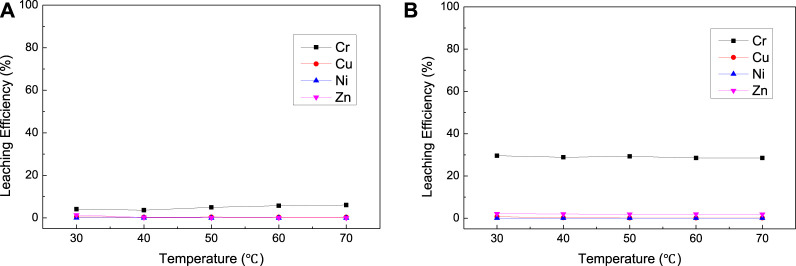
Effect of temperature on the leaching results [**(A)** with H_2_O_2_; **(B)** with NaClO] for complex sludge (EPS2).

The effects of oxidizer dosage, NaOH dosage, and temperature on the leaching efficiency of Cr, Ni, Cu, and Zn from mixed suldge (EPS2) were investigated as shown in Figures 7,8,9. It is interesting that NaClO exhibited stronger leaching ability than H_2_O_2_ for the oxidation leaching of Cr from EPS2, which is contrary to the leaching charateristic of EPS1. The reason might be that H_2_O_2_ can react more easily with the organic matter in the mixed sludge and cause severe decomposition, while NaClO is more stable (Li et al., 2020). The organic matters in the mixed sludge mainly come from the biochemical sludge and oil removal sludge produced during the wastewater treatment stage, and the test results proved that the organic matter content of EPS2 was much higher than that of EPS1, as shown in Table 1. This explanation can also be supported by the experimental phenomena that the reaction is violent and a large amount of bubbles formed when H_2_O_2_ was added to the mixed sludge, indicating that hydrogen peroxide was decomposed quickly.

On the contrary, NaClO exihibited better leaching ability for Cr and good selectivity against Cu, Ni, and Zn. The experimental results show that the oxidation leaching process can effectively extract chromium from EPS, including a significant portion of the residual fraction, while the more mobile heavy metal elements (Cu, Ni, and Zn) stay in the insoluble form. Hence, the oxidation leaching process can achieve the selective extraction of chromium, which is difficult to achieve through traditional acid leaching or pyrometallurgical process. However, this requires a large consumption of NaClO; the leaching efficiency of Cr reached 56.82% when the molar ratio of NaClO/Cr reached about 75:1. It was indicated that NaClO was also consumed by the organic matters in the mixed sludge, while the reaction was less violent than H_2_O_2_ and it can still react with Cr and leach it out.

The results of NaOH dosage and temperature condition experiment (Figure 8 and Figure 9) were similar to the experimental results of EPS1, which indicate that proper amount of NaOH is required to ensure the oxidation leaching of Cr and temperature has little effect on the leaching results.

**FIGURE 10:**
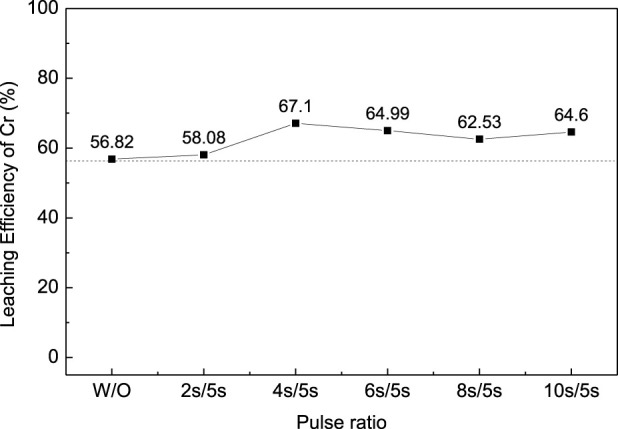
Effect of the ultrasonic pulse ratio on the leaching efficiency of Cr for complex sludge (EPS2).

The oxidation leaching of Cr from EPS2 can also be enhanced by ultrasonication, as shown in Figure 10. Similar trends were observed when the ultrasonic pulse ratio increased as the leaching results of EPS1 (Figure 4). The introduction of ultrasonication can enhance the leaching of Cr from the mixed sludge and reduce the reaction time, and 4s/5s is an appropriate ultrasonic pulse ratio for the optimal leaching result.

**FIGURE 11:**
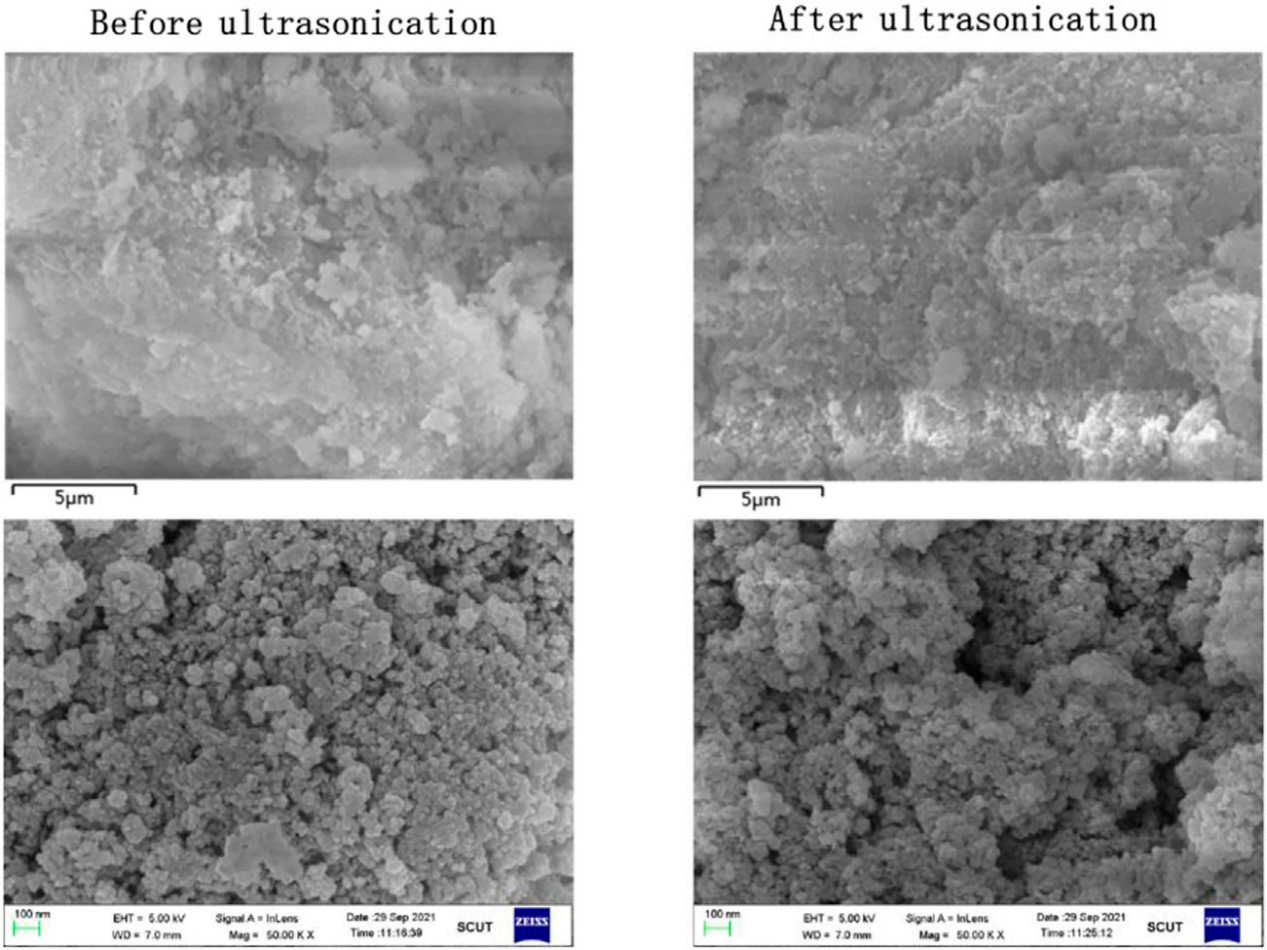
Topography change of complex sludge (EPS2) before and after ultrasonic treatment.

**FIGURE 12:**
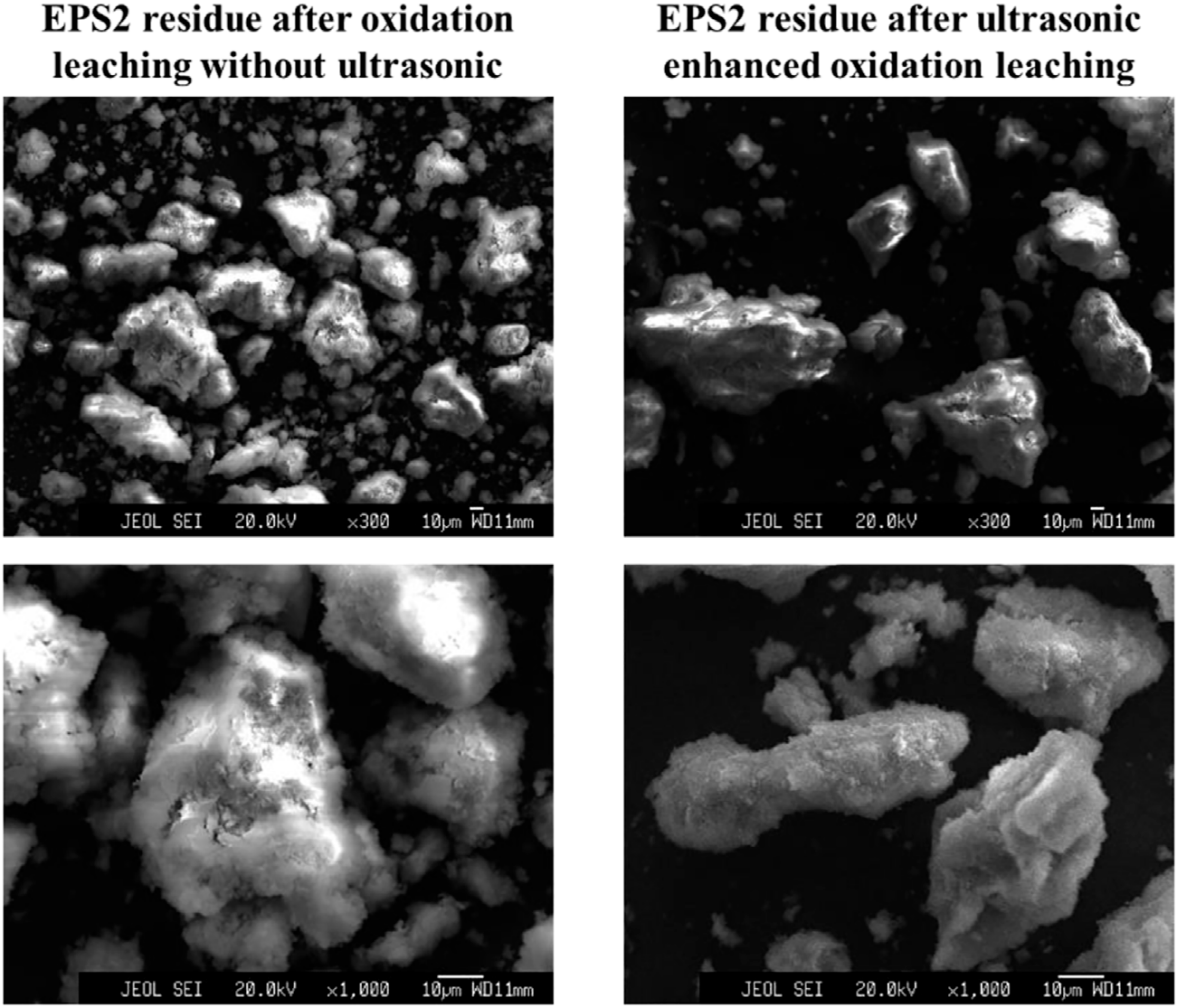
Particle morphology of complex sludge (EPS2) after oxidation leaching with and without ultrasonic treatment.

The electron microscope results of EPS2 (Figures 11, 12) were also similar to those of EPS1. Under the combined effect of particle surface destruction and particle agglomeration, the leaching efficiency of Cr was improved by ultrasonication to a certain extent. Another interesting phenomenon is that the coverings on the surface of the mixed sludge particles seem to be removed after ultrasonicaction, which may facilitate the oxidation reaction on the surface of the sludge particles.

## 4 Conclusion

The oxidation leaching of two different types of Cr-bearing sludge was studied; the experimental results showed that the two kinds of sludge exhibited different leaching characteristics to different oxidizers. H_2_O_2_ exhibited stronger oxidation leaching ability for chromium sludge due to its stronger oxidation capacity and reactivity, whereas it is more susceptible to the influence of organic matter in the mixed sludge. NaClO showed higher leaching efficiency of Cr and good selectivity against other heavy metals in the mixed sludge, while a large consumption of dosage was required. The introduction of ultrasonication can strengthen the oxidation leaching for both kinds of sludges as a result of the combined effect of particle surface damage and particle agglomeration. In addition, ultrasound can also remove the coverings on the surface of the mixed sludge particles, which may facilitate the oxidation reaction.

## Data Availability

The original contributions presented in the study are included in the article/Supplementary Material; further inquiries can be directed to the corresponding authors.

## References

[B1] Arbat A. G. Fesaghandis E. A. Tabrizi A. T. Aghajani H. (2020). Comparison of the effect of NaClO_3_ and H_2_O_2_ on the molybdenum leaching from molybdenite concentrate[J]. Trans. Indian Inst. Metals 73 (9), 2355–2360. 10.1007/s12666-020-02036-1

[B2] Behin J. Akbari A. Mahmoudi M. Khajeh M. (2017). Sodium hypochlorite as an alternative to hydrogen peroxide in fenton process for industrial scale. Water Res. 121, 120–128. 10.1016/j.watres.2017.05.015 28525784

[B3] Carneiro J. Tobaldi D. M. Capela M. N. Novais R. Seabra M. Labrincha J. (2018). Synthesis of ceramic pigments from industrial wastes: red mud and electroplating sludge. Waste Manag. 80, 371–378. 10.1016/j.wasman.2018.09.032 30455018

[B4] Chen H. X. Yuan H. H. Mao L. Q. Hashmi M. Z. Xu F. Tang X. (2020). Stabilization/solidification of chromium-bearing electroplating sludge with alkali-activated slag binders. Chemosphere 240, 124885. 10.1016/j.chemosphere.2019.124885 31568939

[B5] Dai Z. Q. Zhou H. Zhang W. Y. Hu L. Huang Q. Mao L. (2019). The improvement in properties and environmental safety of fired clay bricks containing hazardous waste electroplating sludge: the role of Na_2_SiO_3_ . J. Clean. Prod. 228, 1455–1463. 10.1016/j.jclepro.2019.04.274

[B6] Ding L. Du J. Zhao Y. (2008). Recovery of chromium from sludge by alkaline oxidizing roasting process[J]. Environ. Prot. Chem. Industry 28 (1), 66–69. 10.3969/j.issn.1006-1878.2008.01.016

[B7] Huang R. Huang K. L. Lin Z. Y. Wang J. W. Lin C. Kuo Y. M. (2013). Recovery of valuable metals from electroplating sludge with reducing additives via vitrification. J. Environ. Manag. 129, 586–592. 10.1016/j.jenvman.2013.08.019 24036091

[B8] Kul M. Oskay K. O. (2015). Separation and recovery of valuable metals from real mix electroplating wastewater by solvent extraction. Hydrometallurgy 155, 153–160. 10.1016/j.hydromet.2015.04.021

[B9] Li C. Xie F. Ma Y. Cai T. Li H. Huang Z. (2010). Multiple heavy metals extraction and recovery from hazardous electroplating sludge waste via ultrasonically enhanced two-stage acid leaching. J. Hazard. Mater. 178 (1-3), 823–833. 10.1016/j.jhazmat.2010.02.013 20197211

[B10] Li Y. Yang S. Liu D. Yang C. Yang Z. Li H. (2020). Experimental study of shale-fluids interaction during oxidative dissolution with hydrogen peroxide, sodium hypochlorite and sodium persulfate. Appl. Geochem. 113, 104503. 10.1016/j.apgeochem.2019.104503

[B12] Magalhaes J. Silva J. Castro F. Labrincha J. A. (2005). Physical and chemical characterisation of metal finishing industrial wastes. J. Environ. Manag. 75 (2), 157–166. 10.1016/j.jenvman.2004.09.011 15763158

[B13] Mahmoudi D. Tabrizi A. T. Aghajani H. (2021). Study the variation of surface topography & corrosion resistance of Cr-GO nanocomposite coatings by addition of GO nanoparticles. Surf. Topogr. 9 (1), 015025. 10.1088/2051-672x/abe6f3

[B14] Nguyen V. K. Lee M. H. Park H. J. Lee J. U. (2015). Bioleaching of arsenic and heavy metals from mine tailings by pure and mixed cultures of Acidithiobacillus spp. J. Ind. Eng. Chem. 21, 451–458. 10.1016/j.jiec.2014.03.004

[B15] Rauret G. Lopez-Sanchez J. Sahuquillo A. Rubio R. Davidson C. Ure A. (1999). Improvement of the BCR three step sequential extraction procedure prior to the certification of new sediment and soil reference materials. J. Environ. Monit. 1 (1), 57–61. 10.1039/a807854h 11529080

[B16] Shakiba S. Khabbazi N. S. Tabrizi A. T. (2022). Enhancing the electroplated chromium coating for corrosion protection of aluminum by adding graphene oxide[J]. Surf. Eng. Appl. Electrochem. 58 (2), 202–209.

[B17] Spengler J. Jekel M. (2000). Ultrasound conditioning of suspensions – studies of streaming influence on particle aggregation on a lab- and pilot-plant scale. Ultrasonics 38 (1-8), 624–628. 10.1016/s0041-624x(99)00145-6 10829739

[B18] Veglio F. Quaresima R. Fornari P. Ubaldini S. (2003). Recovery of valuable metals from electronic and galvanic industrial wastes by leaching and electrowinning. Waste Manag. 23 (3), 245–252. 10.1016/s0956-053x(02)00157-5 12737966

[B19] Verbinnen B. Billen P. Coninckxloo M. V. Vandecasteele C. (2013). Heating temperature dependence of Cr(III) oxidation in the presence of alkali and alkaline earth salts and subsequent Cr(VI) leaching behavior. Environ. Sci. Technol. 47 (11), 5858–5863. 10.1021/es4001455 23635007

[B20] Wang M. Gong X. Wang Z. (2018). Sustainable electrochemical recovery of high-purity Cu powders from multi-metal acid solution by a centrifuge electrode. J. Clean. Prod. 204 (PT.1-1178), 41–49. 10.1016/j.jclepro.2018.09.020

[B21] Wu P. Zhang L. J. Lina C. B. Xie X. x. Yong X. y. Wu X. y. (2020). Extracting heavy metals from electroplating sludge by acid and bioelectrical leaching using Acidithiobacillus ferrooxidans. Hydrometallurgy 191, 105225. 10.1016/j.hydromet.2019.105225

[B22] Yan K. Liu Z. Li Z. Yue R. Guo F. Xu Z. (2019). Selective separation of chromium from sulphuric acid leaching solutions of mixed electroplating sludge using phosphate precipitation. Hydrometallurgy 186, 42–49. 10.1016/j.hydromet.2019.03.013

[B23] Yang Y. Liu X. Wang J. Huang Q. Xin Y. Xin B. (2015). Screening bioleaching systems and operational conditions for optimal Ni recovery from dry electroplating sludge and exploration of the leaching mechanisms involved. Geomicrobiol. J. 33 (3–4), 179–184. 10.1080/01490451.2015.1068888

[B24] Yue T. Niu Z. Hu Y. Han H. Lyu D. Sun W. (2019). Cr(III) and Fe(II) recovery from the polymetallic leach solution of electroplating sludge by Cr(III)-Fe(III) coprecipitation on maghemite[J]. Hydrometallurgy 184, 132–139. 10.1016/j.hydromet.2018.11.013

[B25] Zhang C. Song J. Zhang J. Xing J. Hu D. (2018). Understanding and application of an electroplating sludge-derived catalyst with an active texture for improved NO reduction. Sci. Total Environ. 631/632, 308–316. 10.1016/j.scitotenv.2018.02.290 29525710

[B26] Zhang D. Li M. Gao K. (2017). Physical and chemical mechanism underlying ultrasonically enhanced hydrochloric acid leaching of non-oxidative roasting of bastnaesite[J]. Ultrasonics Sonochemistry, 774–781. 10.1016/j.ultsonch.2017.05.020 28733006

[B29] Zhang L. Zhou W. Liu Y. Jia H. Zhou J. Wei P. (2020). Bioleaching of dewatered electroplating sludge for the extraction of base metals using an adapted microbial consortium: Process optimization and kinetics[J]. Hydrometallurgy 191, 105227. 10.1016/j.hydromet.2019.105227

[B27] Zheng J. Lv J. Liu W. Dai Z. Liao H. Deng H. (2020). Selective recovery of Cr from electroplating nanosludge *via* crystal modification and dilute acid leaching. Environ. Sci. Nano 7, 1593–1601. 10.1039/d0en00196a

[B28] Zhou C. L. Ge S. F. Yu H. Zhang T. Cheng H. Sun Q. (2018). Environmental risk assessment of pyrometallurgical residues derived from electroplating and pickling sludges. J. Clean. Prod. 177, 699–707. 10.1016/j.jclepro.2017.12.285

